# AN EXAMINATION OF MEDICATION UTILIZATION DURING MEDICARE HOSPICE ELECTION: A NATIONAL STUDY

**DOI:** 10.1093/geroni/igac059.160

**Published:** 2022-12-20

**Authors:** Thomas Christian, Michael Plotzke

**Affiliations:** Abt Associates, Cambridge, Massachusetts, United States; Abt Associates, Saint Louis, Missouri, United States

## Abstract

In addition to drugs received through the Medicare hospice benefit, each year Medicare hospice beneficiaries receive nearly $700 million in medications through Medicare Part D. Little is known about the nature of these medications. To explore this issue, we identified all beneficiaries who were enrolled in Part D and elected hospice during Calendar Year (CY) 2020. We cross-walked hospice dates of service to Part D medication fill dates. We also collected information on drugs hospices themselves provided and voluntarily reported on hospice claims. Nationally, 1.2 million out of 1.8 million beneficiaries had some drugs provided, through hospice alone (406,423), Part D alone (412,810), and also from both sources (348,659). Medications from Part D tended to be more expensive, upwards of $40 per fill, compared to medications from hospices, which were about $15 per fill or less. Moreover, we found that 98,561 of beneficiaries (8.4%) received the exact same drug from both sources. Across diagnoses, the daily rates of pre-hospice Part D utilization were nearly identical to Part D utilization after electing hospice, and there was no relation to prior Part D use and hospice election duration. Categorically, the greatest amount of Part D expenditures during hospice are for diabetic therapies ($100.2 million), followed by anticoagulants ($97.8 million) and bronchodilators ($57.2 million). CMS should continue to monitor the provision of medications during hospice to maintain the integrity of the benefit and to ensure beneficiaries receive adequate care.

